# Exploring Evolutionary Adaptations and Genomic Advancements to Improve Heat Tolerance in Chickens

**DOI:** 10.3390/ani14152215

**Published:** 2024-07-30

**Authors:** Ali Hassan Nawaz, Phatthawin Setthaya, Chungang Feng

**Affiliations:** 1 College of Animal Science and Technology, Nanjing Agricultural University, Nanjing 210095, China; ah93163@gmail.com; 2 Multidisciplinary Research Institute, Chiang Mai University, Chiang Mai 50200, Thailand; phatthawin.l@cmu.ac.th

**Keywords:** chicken, heat tolerance, domestication, GWAS, QTL

## Abstract

**Simple Summary:**

This study provides a historical perspective of climate change in relation to chickens and a review of genetic advancements related to heat tolerance in chickens. It highlights the origin of the chickens and how the process of domestication and selective breeding has resulted in genetic variations and increased vulnerability to heat stress. This review also discusses the protective genes and molecular mechanisms, such as heat shock proteins, antioxidants, and immunological response that contribute to heat stress tolerance. Likewise, it discusses the use of further genomic techniques such as the genome-wide association study (GWAS) and quantitative trait locus (QTL) analysis in the identification of genetic variables that contribute to the breeding of broiler chickens with enhanced heat tolerance. Similarly, it highlights the need for including local chickens in breeding programs to enhance genetic diversity. This would help them to better withstand heat stress, addressing the challenge of sustainable poultry production in the context of global warming.

**Abstract:**

Climate change poses a significant threat to the poultry industry, especially in hot climates that adversely affect chicken growth, development, and productivity through heat stress. This literature review evaluates the evolutionary background of chickens with the specific genetic characteristics that can help chickens to cope with hot conditions. Both natural selection and human interventions have influenced the genetic characteristics of the breeds used in the current poultry production system. The domestication of chickens from the Red junglefowl (*Gallus gallus*) has resulted in the development of various breeds with distinct genetic differences. Over the past few years, deliberate breeding for desirable traits (such as meat production and egg quality) in chickens has resulted in the emergence of various economically valuable breeds. However, this selective breeding has also caused a decrease in the genetic diversity of chickens, making them more susceptible to environmental stressors like heat stress. Consequently, the chicken breeds currently in use may possess a limited ability to adapt to challenging conditions, such as extreme heat. This review focuses on evaluating potential genes and pathways responsible for heat tolerance, including heat shock response, antioxidant defense systems, immune function, and cellular homeostasis. This article will also discuss the physiological and behavioral responses of chicken varieties that exhibit genetic resistance to heat, such as the naked neck and dwarf traits in different indigenous chickens. This article intends to review the current genomic findings related to heat tolerance in chickens that used methods such as the genome-wide association study (GWAS) and quantitative trait loci (QTL) mapping, offering valuable insights for the sustainability of poultry in the face of global warming.

## 1. Introduction

Heat stress adversely affects the health of domestic animals, resulting in a decline in both animal welfare and economic efficiency. Various domestic animal breeds have obtained resilience and are well adapted to the harsh environments through artificial and natural selection over time [1,2]. Therefore, domestic animals are excellent models for genetic studies and the identification of mutations underlying evolution and adaptation to different environmental conditions. Due to the convenience and simple requirements of feeding conditions during migration, the domestic chicken is closely related to human activity tracks and has become the most diversified domestication model among domestic animals. Selection is responsible for changes in specific genomic regions called “signatures of selection”, which have a role in traits related to morphology, production, immune response, and adaptation to different environments [3,4]. Investigating the presence of signatures of selection is important to better understand the evolutionary history of livestock populations and the genetic mechanisms that underly phenotypic differentiation. Moreover, signatures of selection can be used to identify genes that exert an advantage for a certain population living in specific region [5]. 

Chickens are extremely vulnerable to heat stress because of their homoeothermic nature, which reduces overall productivity, growth, reproductive health, and feed efficiency. The complete understanding of evolutionary adaptations and genetic pathways that enhance heat tolerance is mandatory for the development of heat-resilient chickens [6,7]. Chickens with a high feed conversion ratio (FCR) are not efficient in terms of productivity, which leads to significant financial losses. According to studies, the reduction in feed efficiency of chicken by 1% has the potential to result in an annual cost of up to USD 50 million for global chicken production. However, heat stress results in economic damage up to USD 2.36 billion annually in the United States chicken farming industry [8,9]. Heat stress is also a potential threat to food security, which is a fundamental requirement for human survival. There is growing awareness among the public related to food quality, and its correlation with heat stress especially in the poultry industry needs to be addressed [10,11]. 

Chickens have undergone evolutionary changes from Red junglefowl (*Gallus gallus*), resulting in the development of multiple breeds that have been selectively bred to fulfil different human needs. The deliberate process of selectively breeding chickens to achieve certain traits, such as high meat yield, superior meat quality, and increased egg production, has greatly influenced the overall genetic makeup of contemporary chicken breeds [12,13]. Nevertheless, the process of selection has resulted in a decrease in genetic variety, potentially influencing the chickens’ response to environmental factors like elevated temperatures. Studies have revealed that several genes and pathways are crucial for chickens to adapt to high temperatures. Some examples of genes include those that have a role in heat shock response, antioxidants, immunological response, and cellular homeostasis [10,14]. Genomic selection by targeting these specific genomic regions or genes can be employed to breed chickens with enhanced heat tolerance. The objective of this review is to provide a comprehensive analysis of the genetic and physiological foundations for the adaptation of chicken breeds to heat stress. Consequently, this review seeks to add to the current literature on the genetic selection of chickens with an ultimate view of improving their capacity to acclimatize to high-temperature environments.

## 2. Evolutionary Background

### 2.1. Evolutionary Origin and Domestication of Chicken Breeds

Chicken domestication likely occurred through a commensal route, where wild birds would search for food in locations near human settlements. The major cause for chicken domestication remains uncertain. Without any proof of domestication for the purpose of consuming meat or eggs, it is likely that early farmers chose to keep them for esthetic, socio-cultural, and/or recreational reasons [15]. Genome-wide investigations provide evidence for domestication occurring approximately 8000 years ago during the Neolithic period [4,16]. This process, facilitated by human interventions, resulted in the development of several strains of chickens. Recent research has sought to ascertain the genetic source of commercial chicken breeds, and published findings have revealed the intricate relationship between natural selection and human selection following the period of domestication [16,17]. Human domestication of animals commenced approximately 8000 years ago through the deliberate selection of traits related to egg laying, meat quality, and adaptability [18,19]. This approach of selective breeding resulted in the creation of unique strains that had genetic markers associated with specific traits, particularly basic characteristics related to meat production. In 2023, Wu et al. found that the gene pool of wild Red junglefowl populations has been diminished by 20–50% due to introgression from domestic chickens over the last century [12]. The study compared the whole genome of the chickens and Red junglefowl from two time points and found that the process of domestic introgression has progressively intensified across the Anthropocene. It also identified genes that may have been involved in the domestication of chickens [12]. A recent study examined 863 genomes of junglefowl and indigenous chicken breeds, which indicated that after domestication of chickens, they were moved to Southeast and South Asia, where they mated with genetically distinct local Red junglefowl subspecies and other species of junglefowl. Domestic chickens in China, Southeast Asia, and South Asia now all possess hybrid genomes that derive up to 22.4% of their genetic makeup from Red junglefowl subspecies [13]. 

Indigenous and locally adapted chicken breeds from tropical and subtropical countries exhibit significantly enhanced heat tolerance compared to commercial birds due to the specific environmental constraints they have experienced in their natural habitats. Several studies reported that African indigenous poultry breeds have remarkable adaptability to tough tropical conditions, including prolonged desert seasons, high rainfall and humidity, and extreme heat. Moreover, they demonstrate remarkable resilience against many disease threats. Scientists have identified multiple potential genes and pathways that could be accountable for heat tolerance in chickens through the use of linked and positional cloning investigations [14]. These pathways include heat shock response, oxidative stress response, immunological response, and maintaining cellular homeostasis [3,20]. 

Likewise, crossbreeding of domesticated chicken with wild chicken lines like grey junglefowl has enhanced the capacity of the genotypes of the contemporary chicken breeds [21]. This has enabled them to be more resilient in face of environmental challenges such as heat stress [14,22]. This flow of genes has not only transformed the body and metabolic features of indigenous chicken breeds but also has armed them with desirable genes to cope with different environmental stressors. Understanding the genetic mechanisms or heat tolerance in indigenous chicken breeds and applying sound techniques of selective breeding could help to address the issue of heat stress in the poultry industry [14,23].

### 2.2. Natural Selection and Environmental Adaptations

Through natural selection, various types of chicken breeds have emerged in a way that allows them to live in different environmental conditions. Hence, genetic variability obtained due to random mutations that has spread among different chicken populations helps in augmenting a genetic reservoir of traits influencing thermoregulation and metabolic responses. Heat stress lowers the chances of survival and reproduction in chickens, but the birds that possess a particular mutation with genes to help them endure heat are more likely to live and reproduce successfully. This in turn over several generations of the population alters the distribution of genetic factors within this population and increases the number of genes which provide heat tolerance [24,25]. Research conducted on indigenous chickens identified various potential genes that may be involved in adaptation, particularly in relation to thermotolerance and immunological response. These genes include *cytochrome P450 2B4*, *TSHR*, *HSF1*, *CDC37*, *SFTPB*, *HIF3A*, *SLC44A2*, and *ILF3* [3]. 

Another study on native Italian chickens found that areas under selection included potential genes with biological roles involving disease resistance, immunological responses, and environmental stress, indicating local adaptation of these chicken populations [26]. We can infer that the positive selection observed in Italian chicken populations may have been influenced by the necessity to thrive in given circumstances. Another study on Ethiopian village chickens found special genetic markers linked with heat tolerance in indigenous chicken populations [25]. Such studies show that chicken populations living in hot areas have acquired unique mechanisms to cope with heat stress [27]. Indigenous chicken breeds, on the other hand, are mostly reared as backyard chickens as compared to commercial chicken breeds. Therefore, it can be concluded that indigenous chickens have gained more ability to withstand stress from the environment. Previous studies indicate that selection can play an important role in shaping signatures of selection in local chicken populations and can be a starting point to identify gene mutations that could have a useful role with respect to climate change [20].

Fayoumi chickens, which were examined in a recently published study, are recognized for their capacity to flourish in a hot and humid climate as compared to Leghorn chickens. This indicates that they have developed physiological adaptations to effectively dissipate the metabolic heat [28]. Research conducted on Tibetan chickens suggests that they have undergone genetic adaptations to thrive in high-altitude environments. These changes may involve genes associated with oxygen transportation and metabolic efficiency [29,30]. According to a recently published study, the poultry population in Africa is mostly composed of local or indigenous chickens appreciated for the unique taste and texture of their meat. However, with ever-increasing population growth, the free-range or backyard poultry farming system is no longer enough to supply people with protein requirements. Crossbreeding local hens with an improved rooster weakens the thermotolerance and disease resistance of the offspring in African areas [31]. These investigations highlight the intricate nature of environmental adaptations in chickens, which are influenced by the persistent pressure of natural selection to enhance survival in different climatic situations. 

### 2.3. Impact of Human Interventions or Artificial Selection

Whereas natural selection has contributed significantly to the evolution of heat tolerance in chickens, anthropogenic impacts have also substantially influenced their evolutionary trajectory. Most of the time, selective breeding schemes have focused on the genetic characteristics associated with production traits such as maintaining high egg production in laying chickens and facilitating high growth rates in broiler chickens [32]. Therefore, selective breeding leads to a severe loss of genetic variation of chicken populations. Genetic selection by focusing on particular economic traits, such as high egg or meet productivity, can lead to the elimination of other beneficial genes [33]. The reduction in genetic diversity may render chickens more vulnerable to infections or other environmental stressors for which they have not been selectively selected to resist [31]. Moreover, through the process of selective breeding, it is possible to develop separate breeds of chickens that possess specialized adaptations suited for particular situations [20,34,35]. Likewise, a study on the genome assessment of worldwide chickens has found that commercial chicken breeds used globally for meat and egg production have lost at least half of their ancestral genetic diversity [36]. This study, the first to experimentally analyze genetic diversity across an entire agricultural commodity, highlights significant concerns about the ability of commercial flocks to adapt to future challenges. The poultry industry, which produces over 40 billion birds annually, has seen rapid growth and intensification over the past 50 years, leading to increased inbreeding and a significant reduction in genetic diversity due to a concentrated selection for specific traits like size and egg production [34,37]. Now, breeders are acknowledging the economic benefits of heat tolerance, as demonstrated by varieties such as Fayoumi and naked neck chickens, which are renowned for their capacity to flourish in hot and humid environments. 

The difficulty lies in achieving a harmonious equilibrium between choosing heat tolerance and preserving a certain level of genetic diversity. Heat tolerance can be used as a selection criterion in breeding programs for chickens, along with other important qualities, to guarantee overall resilience [38]. Also, local indigenous chicken breeds, which are genetically well adapted to withstand heat stress conditions in tropical environments, can provide large reservoirs for breeding heat-tolerant traits for the improvement of commercial chicken lines [14,20,39]. 

### 2.4. Comparative Evolutionary Studies

Heat tolerance in some indigenous chicken breeds is due to the adaptability of these breeds and the intricate network of their ancestry, environmental conditions, and genetic makeup. For instance, indigenous breeds from hot and humid climates such as Fayoumi and naked neck chickens have undergone natural selection that made them more adaptable and resilient to prolonged heat exposure [40]. Natural selection has resulted in a variety of physiological changes in these breeds, including smaller bodies in dwarf chickens, frizzled feathers in Kirin chickens, reduced neck feathers in naked neck chickens, and, in certain cases, completely devoid of feathers in scaleless chickens. These adaptations are a result of natural selection pressure that help these chickens to better adapt to the environment by improving heat dissipation efficiency [33,41,42]. Progress in comparative genomics has made it convenient to investigate and detect specific selection signatures in genomes of various breeds. These studies can identify the specific genomic locations that have gone through intense selection pressure. These specific locations may comprise genetic regions that can contribute to heat tolerance by displaying unique adaptation strategies to environmental factors. Several comparative studies among indigenous and commercial chicken lines have revealed specific genetic variants associated with certain traits [2,3,43]. Likewise, a study among indigenous and commercial chicken lines has identified breed-specific single-nucleotide polymorphisms (SNPs) in genes associated with oxidative stress response and heat shock response pathways [44,45]. A recent study has identified 12 genes under positive selection that are involved in adaptations to both tropical desert and tropical monsoon island climates in indigenous chickens from Saudi Arabia and Sri Lanka [46]. Furthermore, another study detected selection signatures in Sri Lankan, Brazilian, and Egyptian chickens, where genes such as *TRMT1L*, *SOCS2*, and *NFKB1* may play a role in the adaptation to hot environments and contribute to their survival [47].

The above discussion and analysis of the comparative evolutionary trajectory of heat tolerance in chickens signifies the role of natural selection in environmental adaptations. An understanding of the foundational genetics behind heat tolerance will enable breeders to devise effective breeding strategies to produce more heat-resilient chickens. By doing so, the genetic diversity of chicken breeds can also be maintained which is important for the long-term survival in face of global climate change. 

## 3. Genomic Studies for Enhancing Heat Tolerance in Chickens

### 3.1. Genome-Wide Association Study (GWAS) and Quantitative Trait Loci (QTL) Mapping

GWAS enables the identification of genetic markers associated with heat tolerance to improve genomic selection technology in chickens. This approach has potential to assist future breeding programs devised to develop heat-tolerant chicken breeds. As such, a GWAS study has identified the multiple genomic regions linked to heat stress response in the F_2_ chicken population [48]; moreover, this study found several genes, including *CEP78*, *MEF2C*, *VPS13A*, and *ARRDC3*, that are responsible for heat resilience. Another study conducted on Nigerian indigenous chickens through whole-genome sequencing data revealed candidate genes/genomic regions under selection for hot climate adaptation, including genes involved in oxidative stress and cellular responses to heat and hypoxia [3]. A recent study investigating the response of a specific chicken breed to Newcastle disease virus found specific regions on chromosomes 1 and 24 that contain genes, namely *KIRREL3* and *ETS1*, which are important for heat stress response in chickens [49]. Furthermore, a comprehensive genomic analysis indicated that the *TSHR* (41020238:G>A) gene variant may enhance the ability to tolerate and adapt to hot environmental circumstances in tropical climes [2]. Numerous significant genes and pathways linked to the genetic adaptation of chickens to tropical desert/monsoon island climates were discovered during a study that examined the selective sweep of the genomes of 67 native breeds of chickens that inhabit hot environments. Particularly, it showed that 12 positively selected genes (PSGs) (*ADCY1*, *CACNA1C*, *CAMK2D*, *PACRG*, *PARK2*, *PRKCH, SDHD*, *SIRT1*, *WNT7B*, *TBXAS1*, *IL18*, and *VPS13C*) were involved in the adaptation of chickens to tropical desert environments [46].

Quantitative trait loci mapping corroborates GWAS by the precise identification of genomic regions that may linked to heat stress response. The identification of QTLs that are systemically present throughout genomes might prove helpful in genomic selection for heat tolerance in chickens [50]. Research has demonstrated that many QTLs are associated with various physiological and production characteristics when subjected to heat stress (Table 1). These QTLs have been identified through the process of QTL mapping and are situated on distinct chromosomes. These factors encompass body temperature, body weight, breast yield, and immunological response. QTLs located on chromosomes 1, 5, and 24 have facilitated the identification of genes associated with oxidative stress, hypoxia response, and thermoregulation. Some examples of these potential genes are *TSHR*, *HSF1*, and *CDC37*. These findings demonstrate the interrelated genetic structure underlying the mechanisms by which heat tolerance is attained through angiogenesis and the augmentation of immune activity [28,50]. Thus, the utilization of QTL mapping in conjunction with genomic selection accelerates the process of generating heat-resistant chicken lines. This can be achieved by carefully choosing particular sections of the genome and candidate genes in order to improve the ability of an individual to withstand high temperatures [51]. 

### 3.2. Genomic Selection and Its Application

**Figure 1:**
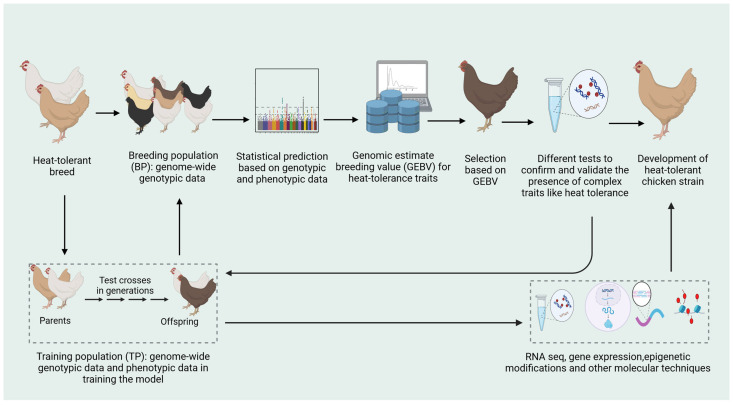
Diagram illustrates the step-wise process of genomic selection for heat tolerance in chickens. Figure was created by using BioRender (https://www.biorender.com/).

Genomic selection is a promising approach for improving heat tolerance in chickens. Genomic prediction models help to identify genetic markers linked with thermotolerance which will be used to selectively identify individuals with heat tolerance-associated traits involved in superior heat tolerance (Figure 1) [14,48]. This innovative approach tackles the future of chicken production, specifically in regions coping with pressing issues of heat stress caused by climate change. High-density genotyping arrays are employed for the examination of millions of single-nucleotide variations across the whole chicken genome. SNPs serve as genetic markers, and their correlation with heat tolerance-related characteristics can be utilized to develop genetically better poultry breeds. The availability of SNP genotyping arrays and advancements in statistical analyses have facilitated the detection of genomic areas and genes that have experienced positive selection in chickens. Various methods have been suggested for detecting signs of selection, including statistical techniques that rely on linkage disequilibrium (LD), variations in allele frequency, regions of homozygosity, and haplotype structure [20]. Recombination seldom happens during the rapid rise of a haplotype carrying a beneficial mutation. Therefore, an ongoing or incomplete indication of selection will have a high-frequency haplotype with extensive linkage disequilibrium. The use of relative extended haplotype homozygosity (EHH)-derived statistics is more effective than single-allele frequency techniques in accurately identifying locations with higher levels of homozygosity [2]. Three commonly used EHH-derived statistics are the integrated haplotype score (iHS), the standardized log ratio of the integrated site-specific EHH between pairs of populations test (Rsb) and the cross-population EHH test (XP-EHH). Selection also causes a decrease in genetic diversity in certain parts of the genome, leading to the formation of successive homozygous genotypes called runs of homozygosity (ROH) islands. Previous studies have demonstrated that ROH islands can serve as a means to identify specific areas of the genome that have an impact on productivity or adaption [20,56].

Several studies have demonstrated that heat shock protein 70 in poultry can serve as a reliable biomarker for identifying chickens with a strong genetic inclination toward heat tolerance in tropical circumstances [58,59]. In addition, a thorough analysis demonstrated that the genes *CEP78*, *MEF2C*, *VPS13A*, and *ARRDC3* are associated with the ability of chickens to survive under conditions of heat stress [56]. Similarly, genomics has demonstrated the ability to enhance heat tolerance in abalone [60] and revealed signatures of selection in different populations of largemouth bass [61] within the field of aquaculture. In a recent study on abalone, a total of 1120 individuals were assessed for their heat tolerance and genotyped using 64,788 quality-controlled SNPs. The heredity of heat tolerance was determined to be of modest magnitude, and the predictive accuracy using BayesB was superior to that of GBLUP. Utilizing genomic selection is a viable method to enhance the heat resistance of abalone [60]. This knowledge can be applied to the field of chicken breeding. In fact, it has also been utilized in the improvement of heat tolerance in dairy cattle through the application of genomic projected breeding parameters. The relationship between the heat tolerance of dairy cow and their heat-resistant qualities serves as helpful insight for poultry breeding endeavors [62]. Another study investigated the expression levels of *HSP90B1* in chicken hypothalamus raised under heat stress settings, indicated that higher expression of *HSP90B1* was associated with heat tolerance [63]. This information equips the breeder with the essential knowledge needed to make intelligent decisions that will result in the production of chickens that are highly resistant to challenges posed by heat stress.

Genomic selection offers a significant benefit by allowing for the comprehensive examination of the entire chicken genome. The introduction of high-density genotyping arrays has greatly transformed genomic selection by enabling the joint investigation of a vast number of SNPs distributed throughout the whole genome [14]. The Affymetrix^®^ Axiom^®^ array is the first commercially available SNP genotyping array for chickens. The array is expected to be utilized in several research and practical applications in the fields of science and in the poultry industry. These applications include genomic selection, genome-wide association studies, selection signature analysis, QTL fine mapping, and CNV identification [64]. The accuracy of genomic prediction using low-density marker panels has been evaluated, showing that high-density panels can achieve up to 95% of the accuracy obtained by using only a small proportion of markers, which is a testament to their efficiency [65]. High-density genotyping arrays, which enable more vast and profound statistical analyses of intensive genomic data, have become an important tool. Hence, in comparison to other methods, the microarray technologies are far better than the traditional methods in that they give the opportunity to study multiple genes at the same time, thus making them the best technology [66,67]. In conclusion, previous literature on the topic has indicated that high-density genotyping arrays are considerably more proficient than conventional methods in genomic selection [68]. Examining the significance of microarray technology and its applications is crucial in understanding the significant transition from conventional methods to the ability of simultaneously analyzing many genes [69,70]. 

## 4. Functional Genes and Candidate Pathways Involved in Heat Tolerance

**Figure 2:**
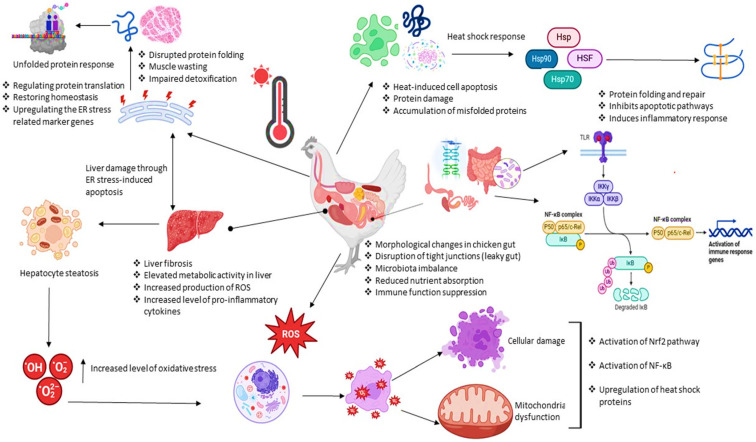
Heat stress triggers different protective pathways and mechanisms in chickens.

Understanding the genetic basis of heat tolerance in chickens is critical for developing breeding programs that can ensure poultry productivity and welfare in increasingly hot climates. Numerous studies have shed light on the functional genes and various protective pathways potentially involved in this complex trait of heat tolerance (Figure 2). Table 2 summarizes previous studies reporting key genes involved in different pathways that play crucial roles in heat tolerance.

The diagram depicts the physiological and cellular responses of chickens to heat stress. It demonstrates the correlation between heat stress and the initiation of the unfolded protein response, heat shock response, as well as the activation of pathways such as Nrf2 and NF-κB. This figure was designed and created in Microsoft PowerPoint (2016). 

### 4.1. Heat Shock Proteins (HSPs)

Heat shock proteins and other molecular chaperones play a crucial function in safeguarding cellular proteins from damage caused by shear stress. Heat shock proteins have a role in the cellular response of chickens to heat stress, and the expression of *HSP70*, *HSPH1*, *HSPA1A*/*HSPA1B*, and *HSP90AB1* is increased in chickens when they experience heat stress damage [58]. To prevent cell death and promote cell survival under heat stress, HSPH1 expression levels increase. A member of the HSP family, *HSP25*, prevents the accumulation of protein-folding intermediates, which in turn prevents apoptosis and maintains the integrity of the cytoskeleton. *BAG3* and *RB1CC1* are linked to the suppression of programmed cell death and apoptosis. By preserving the stability of Bcl-2 family proteins, *BAG3* is activated by *HSF1* and contributes significantly to the survival of cancer cells in situations when programmed cell death is triggered, as found by Jacobs and Marnett [101]. As a result, we suggest that HSF1 can both maintain cellular viability and increase *BAG3* synthesis when temperatures are high. These proteins have a role in the production of the cellular matrix and provide protection against damage caused by stress. Heat shock proteins collaborate with other molecular chaperones to maintain protein and cellular functional integrity during heat stress [102,103]. *HSP70* and *HSP90* are the primary heat shock proteins in chickens that provide protection against heat stress damage, among the heat shock proteins that have been examined. *HSP70* has been identified as the most effective biological marker among the HSPs as it accurately assesses heat stress in chickens. The molecular chaperones *HSP70* and *HSP90* have been reported to interact with other cellular components in order to assist in the folding of proteins and maintaining cellular homeostasis [104,105]. *HSP70*, in conjunction with *HSP90* and *HSP40,* forms a complex that facilitates the extraction of proteins from a cellular compartment. The negative responses also provide compelling evidence of the link between *HSP70* and *HSP90,* which are heat shock factors involved in the transcription of *HSF1,* a crucial regulator of *HSPs* [58,98,106].

Heat shock proteins closely monitor the intricate process of protein folding, unfolding, and refolding to guarantee the survival of cells. In addition, they interact with other chaperones such as *HSP90* and *HSP40* to form complexes that facilitate the process of protein folding. They engage with an essential participant in the heat shock response, known as heat shock transcription factor 1 [58,107]. Heat shock proteins safeguard cells from heat-induced apoptosis by enhancing cytoprotective networks, fortifying them against abrupt temperature changes without necessitating their demise. Moreover, they maintain the equilibrium levels of oxygen in organisms by regulating hypoxia-inducible factor-1, which is a vital component in the control of oxygen balance. Heat shock transcription factors control the expression of HSP genes by preferentially binding to certain sections of the gene, which leads to the activation of gene expression. The combined action of the three heat shock proteins, chaperones, and heat shock factors protects chickens from elevated temperatures, enabling their survival and productivity in challenging environmental circumstances [58,102,108].

The identification of specific genomic areas in the chicken genome that are associated with the production of *HSP70* can be significant in chicken breeding. Such genomic locations can serve as important selection markers in breeding programs to facilitate the improvement of heat tolerance in chickens. These breeding sites can be chosen to increase the expression of *HSP70s*, which are essential for the optimal functioning of cells and protection against various stressors, such as heat stress. *HSP70* has been verified as a prominent biological indicator of heat stress among the investigated HSPs [109]. The choice of these specific areas provides a method for selecting “heat-tolerant” chickens with increased *HSP70* expression. Heat-resistant chickens exhibit an enhanced output due to reduced stress and greater survival rates, making them a safe and advantageous choice for husbandry. Essentially, pinpointing specific regions in the genome that are linked to spontaneous *HSP70* expression offers a distinct opportunity to enhance breeding selection and develop solutions that improve chicken productivity and tolerance to heat stress [58].

### 4.2. Genes Associated with Metabolism and Vascular Function

Genes related to vascular and metabolic function have been shown to be associated with heat adaptation on a genetic level. The genes *FABP2*, *RAMP3*, and *SUGCT* are associated with the physiological response of chickens to heat stress. Therefore, they may enhance the use of energy and perhaps contribute to the metabolic traits found in tropical ecosystems. Furthermore, there is a clear correlation between the ability to withstand high temperatures and a particular genetic alteration known as a missense mutation in the gene responsible for producing the *thyroid-stimulating hormone receptor* (*TSHR*). The *Thyroid-stimulating hormone* (*TSH*) is crucial for regulating metabolic balance in the body when exposed to high temperatures. Furthermore, it appears that *CAMK2* plays a role in controlling the widening of blood vessels and the contraction of smooth muscles, which in turn helps in regulating body temperature by promoting sufficient blood circulation [2,14].

*FABP2*, *RAMP3*, and *SUGCT* play crucial roles in the chicken’s ability to respond to heat stress. They play crucial roles in regulating energy balance and maintaining metabolic stability. *FABP2* transports the majority of the body’s long-chain fatty acids, which are taken up from the intestine into the enterocytes and then metabolized. During periods of heat stress, the function of *FABP2* is very crucial. *RAMP3*, as a constituent of the CLR (calcitonin receptor-like receptor) complex, participates in vascular and metabolic processes. It improves metabolic efficiency by improving food utilization during heat-induced metabolic activity. *SUGCT* also participates in the catabolism of lysine and tryptophan, contributing to the tricarboxylic acid (TCA) cycle. The catabolism of lysine and tryptophan plays a role in the metabolic adaptability necessary for energy production. Consequently, they assist poultry in optimizing nutrition absorption and enhancing their performance under high temperatures [110,111]. Genes related to energy metabolism genes primarily control the absorption and utilization of energy from the food, enabling effective metabolism to fulfil the elevated energy requirements for heat regulation under heat stress circumstances.

*CAMK2* and *TSHR* play key roles in the heat tolerance system of chickens. Both play a part in the physiological reaction to heat stress. The contractility of the vascular smooth muscle is regulated by different isoforms of *CAMK2*. It enables the modulation of blood flow and, consequently, thermoregulation. Vasodilation in birds helps dissipate excess heat and maintain their core body temperature within a specific range, which is particularly crucial during periods of high temperatures. Recently, there have been data indicating that *CAMK2D* exhibits positive selection in chicken breeds living in hot environments, highlighting its vital role in heat adaptation. The second gene is known as the *TSH* receptor gene, or *TSHR*. Its primary function is to control the production of thyroid hormones, which in turn significantly impacts the fundamental metabolic processes responsible for regulating body temperature. A missense mutation in *TSHR* plays a crucial role in maintaining chickens’ metabolic rate and counteracting the negative impact of increasing ambient temperatures, resulting in heat tolerance. Consequently, some indigenous chickens employ this gene to regulate metabolic rate and responsiveness in high ambient temperatures. The combined action of *CAMK2* and *TSHR* results in a complex response that enables birds to not only survive physiological challenges but also enhance their performance under thermal conditions [2,19,112]. Studies have shown that improved circulation in the peripheral blood vessels leads to an increase in the dissipation of heat from the body. The presence of the *CAMK2D* gene, which has undergone positive selection, indicates that chickens from tropical locations adapt to heat stress by regulating blood pressure to dissipate excessive heat and maintain their core body temperature [2].

### 4.3. Genes Linked to Energy Metabolism and Immune Response

Previous studies have emphasized the importance of the energy metabolism-related genes in achieving or maintaining efficient metabolism during heat stress conditions in chickens. *GLUT2*, *FABP1*, *CD36*, *FGA*, *LOXL2*, *GINS1*, and *RRM2* have been documented in the uptake and utilization of energy, and as such, their contribution to metabolic reprogramming is significant [14]. A study conducted on broiler chickens revealed that expression levels of GLUT-2, FABP1, and CD36 were significantly decreased by heat exposure [113]. Indeed, these genes cause the efficient utilization of nutrients in the diet to produce the energy required for the body to maintain homeostasis, while ensuring that the high energy demands associated with heat regulation are met [114,115]. The identification of these genes emphasizes the complex metabolic pathways that are triggered in response to heat stress, allowing chickens to adapt their energy metabolism to cope with the challenges presented by elevated temperatures.

Additionally, studies have shown that heat stress affects not only metabolic genes but also genes related to the immune response, such as *HS3ST5*, *NFAT5*, and *PDK* [14]. *HS3ST5* has a role in the production and breakdown of heat shock proteins, which help protect cells from the effects of heat stress. By regulating apoptosis, it also influences the immunological response [116]. *NFAT5* has a role in modulating the immune response and inflammation. This suggests that it may influence the immune system during periods of heat stress by modulating the pathways associated with inflammation and the stimulation of immune cells [117]. Furthermore, PDK facilitates in preserving energy balance and cellular metabolism which is crucial to enhance immune activity under high environmental temperatures [118,119]. These genes demonstrate the complex interplay between the cellular stress response, immune system, and metabolic adaptations. These interactions are crucial for chickens to maintain internal balance and thrive under stressful circumstances.

### 4.4. Antioxidant Defense Pathway

The antioxidant defense pathway is considered to be one of the most important pathways for heat tolerance in chickens due to the crucial roles played by certain genes and enzymes. Superoxide dismutase is crucial for protecting cells from oxidative damage caused by high temperatures. SOD is composed of many isoforms, including SOD1, SOD2, and SOD3 [120]. Thus, SOD enables the conversion of superoxide radicals to easily disposable oxygen and hydrogen peroxide. Glutathione peroxidase is an enzyme added to the reduction of hydrogen peroxide and organic hydroperoxides. It works in isoforms, which include GPx1, GPx4, and GPx7. This protective process employs a cofactor, glutathione. Catalase, in line with GPx, decomposes hydrogen peroxide to water and oxygen effectively; thus, it protects the cell from being damaged in heat stress conditions [121,122,123]. 

Moreover, the wide range of glutathione S-transferases (GSTs), including GSTA, GSTM, and GSTP isoforms, play a vital role in neutralizing reactive oxygen species and lipid peroxidation products. This functions to protect cells from oxidative damage in the context of heat stress [7,111]. Also, the antioxidant defense pathway exhibits adaptability by safeguarding against oxidative stress induced by many environmental conditions, including low temperatures, illnesses, and pollutants, in addition to its capacity to endure high temperatures [7,14,38]. The antioxidant defense pathway contributes to heat tolerance in chickens by neutralizing reactive oxygen species through the action of enzymes like superoxide dismutase, catalase, and glutathione peroxidase, regulating the expression of antioxidant genes, mitigating oxidative damage to cellular components, and preserving cellular homeostasis under heat stress conditions. Several studies have reported modulation of antioxidant enzyme activities and the beneficial effects of dietary antioxidant supplementation in enhancing the antioxidant defense capacity and improving heat tolerance in poultry [124,125].

### 4.5. Unfolded Protein Response (UPR) Pathway

The unfolded protein response (UPR) is a cellular process that is activated in response to stress in the endoplasmic reticulum (ER) resulting from an excessive accumulation of misfolded and unfolded proteins. It eliminates the severely misfolded proteins through a process called ubiquitination and proteasome degradation. In addition, it reduces the production of unnecessary proteins and promotes the production of proteins that aid in the folding and degradation of other proteins [126]. A network of crucial genes and enzymes regulates this pathway. PERK, sometimes referred to as protein kinase R (PKR)-like endoplasmic reticulum kinase, functions as a surveillance mechanism situated in the endoplasmic reticulum (ER) to detect the accumulation of misfolded proteins. Upon being triggered by endoplasmic reticulum (ER) stress, PERK initiates the phosphorylation of the translation initiation factor eIF2α, resulting in a significant reduction in cellular protein synthesis [127]. 

**Figure 3:**
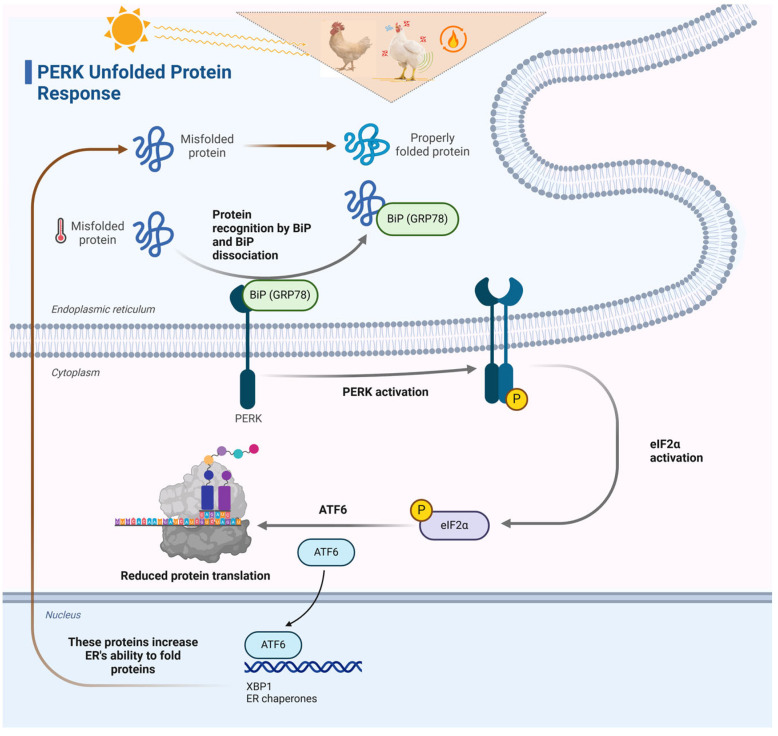
Mechanisms of the unfolded protein response (UPR) in chickens.

*IRE1* and *ATF6* are ER-resident sensors that initiate a cascade of events to enhance the ER’s ability to correctly fold proteins. When activated, IRE1 triggers an unconventional process of cutting and rejoining XBP1 mRNA, leading to an increase in the production of ER-associated protein degradation (ERAD) and ER chaperones. As a result, this enhances the cell’s capacity to manage protein misfolding caused by stress [128]. Upon detection of endoplasmic reticulum (ER) stress, proteolytic enzymes break *ATF6*, leading to its translocation to the nucleus. It increases the transcription of genes that are important for the unfolded protein response (UPR), such as endoplasmic reticulum chaperones, inside the nucleus. Furthermore, the ER-resident chaperone GRP78/BiP (glucose-regulated protein 78/binding immunoglobulin protein) modulates the activation of UPR by regulating the binding state of *PERK*, *IRE1*, and *ATF6*. During periods of endoplasmic reticulum (ER) stress, the separation of these sensors triggers the involvement of GRP78/BiP, which aids in the protein-folding process, hence increasing the ER’s ability to fold proteins [129,130,131]. The unfolded protein response (UPR) pathway, orchestrated by *PERK*, *IRE1*, *ATF6*, and *GRP78/BiP*, is a coordinated response that may alleviate endoplasmic reticulum (ER) stress caused by heat stress in chickens. (Figure 3). This response improves the cells’ capacity to acclimate to and withstand high-temperature surroundings.

This diagram highlights the activation and functioning of PERK, IRE1, and ATF6 in response to ER stress triggered by heat stress and also depicts the regulatory role of GRP78/BiP that enhances the cell’s ability to manage protein misfolding under heat stress. The figure was created by using BioRender (https://www.biorender.com/).

### 4.6. Autophagy Pathway

Autophagy is a cellular process that serves as a defense mechanism and allows cells to adapt. It controls the balance of organisms’ internal environment and serves as a maintenance mechanism for eliminating misfolded or aggregated proteins, clearing damaged organelles like mitochondria, endoplasmic reticulum, and peroxisomes. Moreover, this pathway facilitates the utilization of essential cellular resources during periods of stress [132,133]. The genetic constituents of this system consist of Beclin-1 (BECN1), autophagy-related genes (ATGs), microtubule-associated protein 1 light chain 3 (MAP1LC3/LC3), and mammalian target of rapamycin (mTOR). Each of these genes has distinct functions in the autophagy process [134,135]. During periods of heat stress, the level of activity of BECN1, a crucial controller of the initiation of autophagy, is enhanced. Consequently, autophagosomes are generated, serving a vital function in the autophagy process. Concurrently, additional members of the ATG gene family, including as ATG5, ATG7, and ATG12, are also activated, thereby facilitating the formation and growth of autophagosomes. Moreover, the transformation of LC3-I into LC3-II, a notable marker of autophagosome creation, is considerably increased under heat stress, indicating the active participation of the autophagy pathway [134,135]. 

In addition, heat stress leads to the suppression of mTOR signaling, resulting in enhanced autophagy activation. This results in the deterioration of cellular components and the maintenance of cellular balance. At the molecular level, the onset of autophagy in response to heat stress requires the identification of thermal cues, which then induces the activation of stress-related signaling pathways, namely the heat shock response and oxidative stress response [136,137]. As a result, there is a significant increase in the activation of important genes related to autophagy and the generation of autophagosomes, which in turn enable the breakdown of malfunctioning organelles and proteins. In the end, this coordinated reaction helps to preserve cellular balance, improve tolerance to high temperatures, and facilitate cellular adjustment to thermal stress [136,137]. Therefore, autophagy might play a crucial role as the adaptive mechanism that either protects cellular integrity or aids in cellular death depending on the environmental and cellular conditions. 

### 4.7. NF-κB Signaling Pathway

NF-κB is an essential intracellular signaling molecule that controls the transcription of several genes related to cellular development, inflammatory responses, cell survival, and cell death. The significance of this pathway is highlighted by numerous studies that demonstrate its involvement in various aspects of the stress response. A study revealed that heat stress might induce harm to the pulmonary tissue of broiler chickens by compromising the integrity of the blood–air barrier and increasing permeability. The activation of the TLRs/NF-κB signaling pathways intensifies the inflammatory response, further enhancing this impact and helps chicken to acquire thermotolerance as the duration of heat stress increases, which helps to alleviate the harmful effects caused by heat stress. [138]. Similarly, a study conducted on Ma chickens revealed that heat stress intensifies the inflammation in chickens infected with Escherichia coli O157:H7. Moreover, a study reported the activation of the TLR4-NF-κB signaling pathway which amplified the inflammatory response in the intestine to cope with heat stress [139]. Another study found that heat stress in Salmonella typhimurium-infected broiler chickens leads to a reduction in cytokine production that was possibly triggered by the NF-κB-NLRP3 signaling pathway. These studies highlight the significance of the NF-κB pathway in the regulation of immune response under stress conditions [140]. Additionally, studies have revealed that chronic exposure to heat stress intensifies the NF-κB pathway that accelerates liver inflammation and consequently has a negative impact on the duodenal endothelium through the TLR4-MYD88-NF-κB signaling network [141].

The NF-κB transcription factor is the key element of the NF-κB signaling pathway. It becomes active when its inhibitor, IκB, is phosphorylated and degraded in response to heat stress. Activation leads to the transcriptional regulation of genes involved in immune response, inflammation, and cellular stress adaptability. The activation of the pathway relies on the phosphorylation of IκB, a critical process that is controlled by the IKK complex. The IKK (IκB kinase) complex consists of three components: IKKα, IKKβ, and IKKγ (NEMO). The TNFR superfamily and Toll-like receptors, such as TLR4, are types of activators that increase the NF-κB signaling in response to heat stress. Recent findings suggest that NF-κB and autophagy exhibit an intricate interplay. Therefore, NF-κB has the ability to regulate autophagy processes in order to eliminate damaged cellular components and maintain stability in heat-stressed conditions [139,140,141]. Furthermore, the system enhances cellular ability to adjust and withstand high temperatures by assisting in the regulation of stress response genes, including those that produce heat shock proteins. NF-κB plays a crucial role in integrating different cellular responses to heat stress by interacting with other signaling pathways, including PI3K/Akt and MAPK. This ultimately enhances heat tolerance and promotes cellular survival [142]. 

### 4.8. Mitochondrial Function Pathway

When stress becomes chronic and the cellular response to unfolded proteins in the mitochondria is overwhelmed, the mitochondria can be eliminated by a specific type of cellular self-degradation process known as mitophagy. Mitophagy involves the engulfment of malfunctioning mitochondria into double-membrane vesicles called autophagosomes [143,144]. These autophagosomes then merge with the lysosome, enabling the destruction of the mitochondria. Mitophagy is present at different levels in different types of cells under normal conditions, but it is also triggered in response to stress conditions, such as thermal shock or oxidative stress. Mitochondria are organelles that undergo frequent fusion or fission events, contributing to mitochondrial homeostasis and influencing the formation of contact sites with other cellular organelles. These organelles are highly dynamic in nature [145,146]. Dynamin-related protein 1 (Drp1), which belongs to the dynamin family, plays a crucial role in mitochondrial fission. The reorganization of mitochondria plays a vital part in determining the ability of chickens to tolerate heat [143]. The central element of this system is the master regulator PGC-1α, which orchestrates the generation of new mitochondria by activating the expression of genes essential for duplicating mitochondrial DNA, transcribing it, and building the electron transport chain. Concurrently, the equilibrium between the fusion of mitochondria, regulated by mitofusins (*MFN1* and *MFN2*), and the fission of mitochondria, aided by Drp1, ensures the elimination of impaired mitochondria through mitophagy. This mechanism maintains the functionality and dispersion of mitochondria inside the cell [147,148]. 

Furthermore, the mitochondrial respiratory chain, including cytochrome c oxidase (COX) and NADH dehydrogenase subunits, together with ATP synthase subunits, facilitates the production of ATP, which is essential for fulfilling cellular energy needs during heat stress. The genes SOD2, GPX4, and TXNRD2 play a crucial role in creating mitochondrial antioxidant enzymes. These enzymes actively eliminate reactive oxygen species, thereby protecting mitochondria from oxidative damage and ensuring their proper functioning. In addition, the regulation of mitochondrial calcium levels by MCU (mitochondrial calcium uniporter) and NCLX (mitochondrial sodium calcium exchanger) ensures precise communication and functioning, hence improving the cells’ ability to endure heat stress [147,149,150]. These pathways emphasize the critical significance of mitochondrial function and biogenesis in enhancing cellular adaptation and survival in heat stress conditions. 

## 5. Conclusions

In this literature review, the evolutionary origins and genomic improvements in the heat tolerance of chickens are discussed. Various functional genes and pathways are shown to be involved in cellular signaling under heat stress conditions; such pathways include heat shock proteins, antioxidant defense, unfolded protein response, autophagy, and overall mitochondria functioning. Genomic approaches like genome-wide association studies (GWASs), quantitative trait loci (QTL) mapping, and genomic selection have enabled the discovery and application of genetic markers associated with heat tolerance. While significant progress has been made, several research gaps remain to be addressed. Future studies should further elucidate the complex gene networks and regulatory mechanisms underlying heat tolerance. Integrating multi-omics data and advanced computational methods could provide a more comprehensive understanding. Future research employing advanced genomic editing techniques like CRISPR/Cas9 to manipulate or validate the functioning of candidate genes associated with heat tolerance may be an alternative to improve heat tolerance in chickens [151,152]. In addition, studying various epigenetic mechanisms could also prove helpful to gain valuable insights in order to improve the heat tolerance of chickens [153].

Breeding strategies should aim to balance heat tolerance with preserving overall genetic diversity and production traits. Incorporating genetic resources from indigenous chickens could enrich the heat-tolerant gene pool. Ultimately, developing heat-resilient chicken breeds is crucial for ensuring sustainable poultry production and welfare under the challenges of global climate change. 

### Declaration of Generative AI and AI-Assisted Technologies

During the preparation of this work, the author(s) used generative AI and an AI-assisted tool (Quillbot) to improve the writing process. The tool was employed to improve language clarity and language fluency. After using this tool/service, the author(s) thoroughly reviewed and edited the content, ensuring its coherence and authenticity and made necessary refinements to the content, fully taking responsibility for this publication’s material.

## Figures and Tables

**Table 1:** Recent studies in chicken genomics have identified various genes in specific genomic regions that have crucial roles in heat tolerance.

Trait Influenced by HS	Candidate Genes	Functions	Chicken Strain	References
Body temperature	*USP22*, *KCNH6*, *MYL4*, *MIF*, *ACE*, *DDX42*	Disruption of DNA synthesis, RNA processing and translation, cell signaling, apoptosis, and blood vessel development	Broiler × Fayoumi AIL	[50]
Body temperature	*EZH2*, *RAD21*, *GSK3B*, *MLH1*, *RTEL1*	Apoptosis, cellular stress responses, DNA repair, and metabolic oxidative stress	Broiler-type indigenous chicken	[52]
Body weight	*HHEX*, *MARCH5*, *HECTD2*, *IDE*, *TNKS2*, *KIF11*	Apoptosis, immune response, and DNA synthesis	Broiler × Fayoumi AIL	[50]
Digestibility	*SLC13A5*, *PITPNM3*, *XAF1*, *TXNDC17*	Protein synthesis, membrane permeability, and free radical damage	Broiler × Fayoumi AIL	[50]
Heat tolerance	*TSHR*	Photoperiodic response in chickens	Indigenous chickens in tropical regions	[53]
Heat tolerance and immune response	*HSP70*, *HSPA9*, *HSPH1*, *HSP90AB1*, *PLCB4*	Heat shock response and immune system activation	Iranian indigenous chickens	[20]
Lack of feathers	*FGF20*	Development of spur and feathers in chicken	Scaleless chickens	[54]
Mortality under heat stress	*LRP11*, *HTR2B*, *EIF2B5*, *SOD2*, *DBH*, *ERN1*	Linked to various immune and physiological functions	White Leghorn layer line	[51]
Production, physiological, and egg quality traits	*SSTR2*, *SOX9*, *HRAS*, *IRF7*	Cellular stress response and immune response activation	Commercial white egg-laying hens	[55]
Response to heat stress	*CEP78*, *MEF2C*, *VPS13A*, *ARRDC3*	Heat stress resistance in birds	F_2_ chicken population	[56]
Stress response	*THADA*, *TRPC3*, *MOV10L1*, *RAD51B*, *TGFB2*	Cellular stress response, DNA repair, apoptosis, and metabolic oxidative stress	Indigenous broiler chickens	[57]
Survival to acute heat stress	*VGFR4*, *SLC16A2*, *COX7B*, *AGPAT5*, *HSF2BP*, *SLC35F2*	Genes are linked to ubiquitin-mediated proteolysis, metabolism, and homeostasis.	Commercial layer chickens	[6]
Thermotolerance	*SCEL*, *KCNS2*, *STK3*	Heat stress resistance	Broiler-type strain Taiwan country chickens	[52]
Viral titer under heat stress	*CAMK1d*, *CCDC3*	These genes are responsible for immune response in chicken.	Hy-Line Brown layer chickens	[49]
Viral titer under heat stress	*TIRAP*, *ETS1*, *KIRREL3*, *ST3GAL4*	Adaptive immune response	Hy-Line Brown layer chickens	[49]

USP22: Ubiquitin-Specific Peptidase 22, KCNH6: Potassium Voltage-Gated Channel Subfamily H Member 6, MYL4: Myosin Light Chain 4, MIF: Macrophage Migration Inhibitory Factor, ACE: Angiotensin-Converting Enzyme, DDX42: DEAD-Box Helicase 42, EZH2: Enhancer of Zeste Homolog 2, RAD21: RAD21 Cohesin Complex Component, GSK3B: Glycogen Synthase Kinase 3 Beta, MLH1: MutL Homolog 1, RTEL1: Regulator of Telomere Elongation Helicase 1, HHEX: Hematopoietically Expressed Homeobox, MARCH5: Membrane-Associated Ring-CH-Type Finger 5, HECTD2: HECT Domain E3 Ubiquitin Protein Ligase 2, IDE: Insulin-Degrading Enzyme, TNKS2: Tankyrase 2, KIF11: Kinesin Family Member 11, SLC13A5: Solute Carrier Family 13 Member 5, PITPNM3: Phosphatidylinositol Transfer Protein Membrane-Associated 3, XAF1: XIAP-Associated Factor 1, TXNDC17: Thioredoxin Domain Containing 17, TSHR: Thyroid-Stimulating Hormone Receptor, HSP70: Heat Shock Protein 70, HSPA9: Heat Shock Protein Family A (Hsp70) Member 9, HSPH1: Heat Shock Protein Family H (Hsp110) Member 1, HSP90AB1: Heat Shock Protein 90 Alpha Family Class B Member 1, PLCB4: Phospholipase C Beta 4, FGF20: Fibroblast Growth Factor 20, LRP11: LDL Receptor-Related Protein 11, HTR2B: 5-Hydroxytryptamine Receptor 2B, EIF2B5: Eukaryotic Translation Initiation Factor 2B Subunit 5, SOD2: Superoxide Dismutase 2, DBH: Dopamine Beta-Hydroxylase, ERN1: Endoplasmic Reticulum To Nucleus Signaling 1, SSTR2: Somatostatin Receptor 2, SOX9: SRY-Box Transcription Factor 9, HRAS: HRas Proto-Oncogene, GTPase, IRF7: Interferon Regulatory Factor 7, CEP78: Centrosomal Protein 78, MEF2C: Myocyte Enhancer Factor 2C, VPS13A: Vacuolar Protein Sorting 13 Homolog A, ARRDC3: Arrestin Domain Containing 3, THADA: THADA Armadillo Repeat Containing, TRPC3: Transient Receptor Potential Cation Channel Subfamily C Member 3, MOV10L1: Mov10-Like RISC Complex RNA Helicase 1, RAD51B: RAD51 Paralog B, TGFB2: Transforming Growth Factor Beta 2, VGFR4: Vascular Endothelial Growth Factor Receptor 4, SLC16A2: Solute Carrier Family 16 Member 2, COX7B: Cytochrome C Oxidase Subunit 7B, AGPAT5: 1-Acylglycerol-3-Phosphate O-Acyltransferase 5, HSF2BP: Heat Shock Transcription Factor 2 Binding Protein, SLC35F2: Solute Carrier Family 35 Member F2, SCEL: Sciellin, KCNS2: Potassium Voltage-Gated Channel Modifier Subfamily S Member 2, STK3: Serine/Threonine Kinase 3, CAMK1d: Calcium/Calmodulin-Dependent Protein Kinase ID, CCDC3: Coiled-Coil Domain Containing 3, TIRAP: Toll-Interleukin 1 Receptor (TIR) Domain Containing Adaptor Protein, ETS1: ETS Proto-Oncogene 1, Transcription Factor, KIRREL3: Kin of IRRE-Like 3 (Drosophila), ST3GAL4: ST3 Beta-Galactoside Alpha-2,3-Sialyltransferase 4.

**Table 2:** Pathways and genes identified by various studies that have potential roles in response to heat stress in chickens.

Associated Pathways	Genes Involved	Function	Protective Role in Heat Stress	References
Apoptosis and Cell Survival	*RB1CC1*, *BAG3*	Regulate apoptosis and cellular stress responses	Mitigate cellular apoptosis in the presence of adverse conditions	[71,72]
Behavioral Adaptation	*DRD1*, *DRD2*, *SERT*	Dopamine and serotonin receptors	Affect stress-induced behaviors and feed intake	[73,74,75]
Cellular Signaling	*MAPK*, *JNK*, *ERK*, *PI3K*	Signal transduction pathways	Facilitate cellular adjustments to exogenous stress signals	[76,77,78]
DNA Repair and Integrity	*BRCA1*, *RAD51*, *MSH2*	Involved in DNA damage repair	Ensure genomic integrity and inhibit mutation during periods of stress	[14,79]
Feather Development	*BMP2*, *FGF*, *EDAR*	Involved in feather follicle development	Impact the density and shape of feathers to regulate body temperature	[14,54]
Gut Health and Integrity	*ZO-1*, *OCLN*, *MUC2*	Structural components of gut barrier	Preserve the integrity of the intestinal barrier and enhance the process of absorbing nutrients	[80,81]
Heat Shock Proteins (HSPs)	*HSP70*, *HSPH1*, *HSPD1*, *HSP90AB1*, *HSPB1*, *HSPA8*	Chaperone proteins help in protein folding	Protect cellular constituents from injury and facilitate the process of recovery Their increased expression in response to heat stress is a crucial strategy for protecting and recovering cellular function.	[82,83]
Immune Response and Energy Metabolism	*HS3ST5*, *NFAT5*, *PDK*	Involved in immune response and energy metabolism	Improve the ability of cells to withstand and manage energy during times of stress	[47,84,85]
Lipid Metabolism	*ACC*, *FAS*, *SCD*, *SREBP-1c*, *PPARα*	Enzymes and regulators of lipid synthesis	Under conditions of heat stress, dysregulation results in an elevated accumulation of fat.	[86,87]
Metabolism and Energy Conversion	*GLUT2*, *FABP1*, *CD36*, *FGA*, *LOXL2*, *GINS1*, *RRM2*	Transporters and enzymes for metabolism	Enhance energy generation and maintain metabolic stability under high temperatures Crucial for preserving homeostasis during periods of heat stress	[14,88]
Muscle Development and Growth	*Myostatin*, *Smad3*, *FoxO4*, *MAFbx*, *MuRF1*, *IGF1*, *Akt*, *MyoD*	Regulate muscle protein synthesis and breakdown	Heat stress inhibits muscle hypertrophy and stimulates protein catabolism.	[89,90,91]
Neuroendocrine and Stress Signaling	*CRH*, *POMC*, *AVP*	Hormones and regulators in stress response	Regulate stress reactions and behavior in response to elevated temperatures	[92,93,94]
Oxidative Stress and Detoxification	*SOD1*, *CAT*, *GPX1*	Antioxidant enzymes	Prevent oxidative damage caused by heat stress.	[95,96]
Thermoregulation and Stress Response	*HSF1*, *HSF3*	Heat shock factors regulating HSP expression	Regulate the physiological reaction to stress and enhance the ability to withstand high temperatures.	[97,98]
Thyroid Hormone Activity	*TSHR*	Thyroid-stimulating hormone receptor	Controls the generation of heat and the rate of metabolism.	[2]
Vascular and Muscle Contraction	*MYLK2*, *BDKRB1*	Muscle contraction and blood flow regulation	Ensure adequate circulation and optimal muscular performance in the presence of elevated temperatures. Essential for the dispersion of heat.	[99]
Water and Electrolyte Balance	*AQP1*, *AQP3*, *NKCC1*	Water channels and ion transporters	Maintain proper hydration levels and ensure equilibrium of ions within cells.	[100]

RB1CC1: RB1 Inducible Coiled-Coil 1, BAG3: BCL2-Associated Athanogene 3, DRD1: Dopamine Receptor D1, DRD2: Dopamine Receptor D2, SERT: Serotonin Transporter (SLC6A4 gene), MAPK: Mitogen-Activated Protein Kinase, JNK: c-Jun N-terminal Kinase, ERK: Extracellular Signal-Regulated Kinase, PI3K: Phosphoinositide 3-Kinase, BRCA1: Breast Cancer 1, RAD51: RAD51 Recombinase, MSH2: MutS Homolog 2 BMP2: Bone Morphogenetic Protein 2, FGF: Fibroblast Growth Factor, EDAR: Ectodysplasin A Receptor ZO-1: Zonula Occludens 1, OCLN: Occludin, MUC2: Mucin 2 HSP70: Heat Shock Protein 70, HSPH1: Heat Shock Protein Family H (Hsp110) Member 1, HSPD1: Heat Shock Protein Family D (Hsp60) Member 1, HSP90AB1: Heat Shock Protein 90 Alpha Family Class B Member 1, HSPB1: Heat Shock Protein Family B (Small) Member 1, HSPA8: Heat Shock Protein Family A (Hsp70) Member 8 HS3ST5: Heparan Sulfate Glucosamine 3-O-Sulfotransferase 5, NFAT5: Nuclear Factor of Activated T-Cells 5, PDK: Pyruvate Dehydrogenase Kinase ACC: Acetyl-CoA Carboxylase, FAS: Fatty Acid Synthase, SCD: Stearoyl-CoA Desaturase, SREBP-1c: Sterol Regulatory Element Binding Transcription Factor 1c, PPARα: Peroxisome Proliferator-Activated Receptor Alpha, GLUT2: Glucose Transporter 2, FABP1: Fatty Acid-Binding Protein 1, CD36: CD36 Molecule, FGA: Fibrinogen Alpha Chain, LOXL2: Lysyl Oxidase-Like 2, GINS1: GINS Complex Subunit 1, RRM2: Ribonucleotide Reductase Regulatory Subunit M2, Smad3: SMAD Family Member 3, FoxO4: Forkhead Box O4, MAFbx: Muscle Atrophy F-Box, MuRF1: Muscle RING-Finger Protein-1, IGF1: Insulin-Like Growth Factor 1, Akt: AKT Serine/Threonine Kinase, MyoD: Myogenic Differentiation 1, CRH: Corticotropin-Releasing Hormone, POMC: Proopiomelanocortin, AVP: Arginine Vasopressin, SOD1: Superoxide Dismutase 1, CAT: Catalase, GPX1: Glutathione Peroxidase 1, HSF1: Heat Shock Transcription Factor 1, HSF3: Heat Shock Transcription Factor 3, TSHR: Thyroid-Stimulating Hormone Receptor, MYLK2: Myosin Light Chain Kinase 2, BDKRB1: Bradykinin Receptor B1, AQP1: Aquaporin 1, AQP3: Aquaporin 3.

## Data Availability

Data contained within the article.
